# Understanding medical students’ transformative experiences of early preclinical international rural placement over a 20-year period

**DOI:** 10.1186/s12909-022-03707-x

**Published:** 2022-08-31

**Authors:** Bunmi S. Malau-Aduli, Karina Jones, Amy M. Smith, Tarun Sen Gupta, Richard B. Hays

**Affiliations:** College of Medicine and Dentistry, James Cook University, Townsville, Australia

**Keywords:** Transformational Learning Theory, medical education, preclinical international rural placement, career choice, role models, rural healthcare

## Abstract

**Introduction:**

Rural placements are an important component of rural medical education programs seeking to develop rural practice pathways for medical students. These placements are usually domestic, but James Cook University in Australia developed an international rural placement program in the first half of the medical course that was funded through bursaries. This study explores how the international rural placement helped to shape the lives (personal development and learning) of the participants, using Transformational Learning Theory as a framework for identifying and describing the transformational elements, process and impact of the program.

**Methods:**

Sixty-five students received a bursary for an international rural placement between 2001–2019. All were contacted by email and invited to participate in a short survey and a follow-up interview. Fifteen participants agreed and twelve were able to participate in individual semi-structured interviews which were recorded, transcribed and analysed using inductive thematic analysis.

**Results:**

Participants reported that the bursary provided a “once in a lifetime opportunity” to “experience eye-opening and culturally rich difference”. Nonetheless, some elements of the placement experience presented disorientating dilemmas that triggered deep reflections and shifts in perceptions. The bursary recipients realised that “being open-minded” allowed them “enjoy good company”. They were also able to assume “outsider view which allowed reassessment of their own country” and the “isolation experiences gingered desire to right health wrongs”. The triggers and mental shifts had significant impact on the bursary recipients and fostered the development of “inspirational new horizons” based on an appreciation of the “value of rural practice” and “role-modelling for life-long learning.” These findings are consistent with Transformational Learning Theory.

**Conclusion:**

Participants in this study reported meaningful and strongly positive impacts from the experiences gained during an international rural clinical placement early in their course. They described transformative experiences which appear to contribute strongly to personal development. This finding supports maintaining opportunities for international experiences during rurally-oriented medical programs as these may impact longer term career choice.

**Supplementary Information:**

The online version contains supplementary material available at 10.1186/s12909-022-03707-x.

## Introduction

The global challenges in producing and retaining a rural and remote medical workforce are well documented [1, 2]. The James Cook University (JCU) medical program commenced in 2000 as an entirely regional program with emphasis on the health of rural, remote, tropical and First Nations communities [3]. Several strategies known to positively influence rural career intent were incorporated – selection of rural background students; ‘ruralised’ curriculum and assessment content and process; rural clinical placements, rural community connections; and exposure to strong rural role models and mentors. So far, the outcomes have broadly met with the expectations that such training opportunities will lead to greater intentions to go rural and uptake of rural practice upon completion of medical training [4, 5]. While all medical programs in Australia now implement similar strategies to varying degrees, the JCU program does more on a program-wide basis. For example, all students undertake a minimum of 20 weeks of rural placement over the program in Years 2, 4 and 6 and many opt to do additional placements, potentially up to a total of 100 weeks.

### Organisational context—Intervention

One such initiative was a mandatory, four-week rural community placement in the middle or end of Year 2, designed as part of a sustained focus on understanding rural professional life and practice throughout the program. Like many young adults, students had expressed interest in international experiences and early JCU program leaders had connections with like-minded programs in other countries, particularly in North Dakota (USA) and Tromso (Norway), both with similar health and workforce challenges and substantial First Nations populations. Opportunities were created for additional or longer elective international medical placements that would encourage reflection on international similarities and differences in rural health care. The North Dakota Bursary (later known as the Lynn Kratcha Memorial Bursary) was founded in 2001 by a rural doctor in North Dakota, USA, who had personally undertaken elective placements in North Queensland (Australia) during both his undergraduate and Family Practice residency training. The second was an exchange of students with the University of Tromso that commenced in 2001. Opportunities have since been added in Tennessee (USA) and Saskatchewan (Canada). A total of five (5) funded international medical electives are now available each year for a cohort of about 180 students.

Innovative features of the JCU program include that it is an international elective placement targeted at medical students at a junior level of training. There is a consistent, highly competitive selection process, comprising three-stages: a written application (usually a review of a relevant book or film); an interview which explores the applicant’s suitability to be an ambassador for JCU; and a public oral presentation on a topic relevant to rural medicine, prepared as a potential presentation for an international audience while on placement. The major criteria utilised in the selection process include motivation level as indicated by prior involvement in student group or rural health activities; the ability of the applicants to articulate how their participation in an overseas rural placement would benefit the JCU community and its relevance to rural or remote life/health/Indigenous health; and their commitment to rural career. Usually, between 15–20 students apply for the bursary and five (5) are awarded.

These supported electives are available only to year two medical students and are more community-based and observational than the more intensive, clinical skills-focused placements in senior years. Most participants are young adults (approximately 18–20 years of age). According to Erikson’s theory of psychosocial development and more recent variations, this is a period of major change as young adults develop their own identities [6, 7]. Shifts in attitudes, beliefs, values, and behaviours can be facilitated by removing learners from their routine environment [8], placing them in situations ‘at the edge of their understanding’ [9–11].

A total of 65 students received a Bursary between 2001 and 2019. No bursaries were allocated in 2020–21 due to the COVID-19 pandemic. The current locations of all 65 were identified through the Australian Health Professions Regulatory Authority (AHPRA) public website. As shown in Table 1, while numbers are small, majority of those in both graduated and current undergraduate groups are outside of metropolitan areas as defined under the Modified Monash Model (MM Model). Within the MM Model, there are seven (7) categories based on road distance and the population of cities, towns and communities: MM1, Metropolitan areas; MM2; Regional centres; MM3, Large rural towns; MM4, Medium rural towns; MM5, Small rural towns; MM6, Remote communities; and MM7, Very remote communities [12]. The sole international location is also a rural community.

**Table 1:** Specialty training status and practice location of Rural Bursary holders 2001–2019

MMM Level	1	2	3	4–5	6–7	International	Not traced	Total
Cohort 2006–2015 (Training complete) n (%)	14 (42%)	6 (18%)	1 (3%)	9 (27%)	1 (3%)	1 (3%)	1 (3%)	33 (99%)^a^
Cohort 2016–2019 (In-training) n (%)	5 (31%)	5 (31%)	0	1 (6%)	2 (13%)	0	3 (19%)	16 (100%)
Students n		16						16

^a^Rounding error

### Rationale for this study

The intention of this early, immersive, learning experience, involving travel to a distant land, was to combine an adventurous new experience with encouragement to regard working in a rural location after graduation as interesting and desirable. While this international rural bursary placement program has been popular, little is known about the impacts of this significant investment of time and resources. Medical education is often based on empirical, culture-bound practices rather than theoretical underpinning [13]. Most of the existing literature on student placements focuse on the efficacy of clinical placements occurring later in students’ medical training. There is a paucity of data on the long-term impact of these early placements, especially in the rural context. Twenty years on, we sought to formally explore the experiences of participants and identify any alignment with relevant transformational theory [14]. In doing so, this exploration may improve understanding of those experiences and any influences on both their return to their studies and career choice, contributing to an evidence base for this innovative rural medical education strategy.

### Theoretical context

We chose Transformational (or transformative) Learning Theory as a framework for this study because of its relevance to young adult learning and the potential for adjustment in thinking based on new information [15]. This theory provides a framework for identifying and describing the transformational elements and process that occur for program participants. As described by Mezirow, transformational learning focuses on five major developmental elements, namely: critical reflection on assumptions, use of empirical methods for validation and accuracy (instrumental learning), use of continuing discourse to justify beliefs (communicative learning), taking action on transformed perspective and actioning of transformed insight [15]. Transformational Learning Theory proposes a process for adult learning that goes beyond simply acquiring knowledge to incorporating critical reflection to deconstruct and reconstruct their previously held worldview [16].

The efficacy of the transformational process depends on the motivation/readiness for change [17]. The process begins when the learner faces a “disorienting dilemma” [18, 19], a situation, context, or event/ series of events, that triggers an awareness that they are holding a limited or distorted perspective not appropriate to the particulars of this context [18]. The dilemma triggers a period of critical reflection and self-reflection, through which the learner enhances their understanding of their own frame of reference, its appropriateness to the situation, considers and trials alternative feelings, attitudes, beliefs, priorities, and/or behaviours [20]. Through the trial-and-error process, the learner makes notable changes in the ways in which they interact with the world, via establishing modified roles and relationships [16]. Through enhanced competence and self-confidence in these new roles, the learner reintegrates and repositions themselves into the world, which guides future action. The overall impact of these changes can include shifts in self-understanding, relationships with others, ways of thinking, and overall interpretation of and responses to the external world [16, 18, 21]. The instrumental, communicative and emancipatory elements of learning provide a valuable framework to investigate experiences and connections [17].

Using the Transformational Learning Theory as a framework, this study sought to address the overarching research question: How has the second-year international rural placement helped to shape the lives (personal development and learning) of the Bursary participants? To explore this concept fully, we addressed the following specific questions.

1. What were students’ motivations for applying?
2. What were the transformational elements of the placement experience?
3. How did the participants process their transformative experiences?
4. What was the impact of the placement experience on longer-term learning and clinical practice? (Including return to studies, career specialisation, decisions of where to live and work, and their character/self-identity).

## Methods

### Study design

This qualitative study operated within a constructivist research paradigm, taking a relativist-subjectivist ontological-epistemological perspective [22–24] and utilised semi-structured individual interviews. A phenominological methodology [25] with an inductive thematic analysis approach was employed to explore the participants’ lived experiences of their second-year international rural placement. Furthermore, theoretical framework analysis was utilised in identifying and contextualising the transformative learning aspects of this second-year international rural placement. This research was approved by James Cook University’s Institutional Human Ethics Review Committee (H6921).

### Recruitment strategy

All 65 students who had received a Bursary between 2001 and 2019 were invited to participate by an email to their last known address in university records. A link was included to a SurveyMonkey site that sought demographic information designed to: (a) classify interviewees in terms of their demographics, and (b) provide enough information about the participant to enable the interviewer to prepare interview questions tailored to their experience. All 15 participants who expressed an interest in being interviewed were emailed an invitation to select a suitable interview time and 12 were available for interviews within the timeframe of the study – between October 2020 and January 2021.

### Interview procedure

Individual interviews were conducted by an experienced qualitative interviewer (AS) via a live-streaming software platform. Interviewees chose whether they preferred an audio-only or video interview and only audio-recordings were used in the analysis. Open-ended, semi-structured interview questions surrounding participants’ experiences with the application process, time on placement, return to study, and the role the placement experience played in shaping career choice, where to live and work, and their personal identity were followed up with probing questions to determine whether a transformational experience occurred, identify the trigger, the transformational process, and the factors that influenced the transformational shift (Appendix 1). The interviewer took detailed notes for all interviews, recording key ideas and initial impressions. The notes were analysed as part of the interview process. This allowed for the emergence of themes and the refinement and differentiation of themes to occur through subsequent interviews.

### Data analysis

All interview audio-recordings were transcribed in the electronic software – OTTER and verified by KJ. NVivo version 12 software (QSR International, Melbourne, Australia) was used to assist with data management and analysis. All transcripts were de-identified and analysed by AS, KJ and BMA, with discrepancies resolved in a consensus meeting. Data analysis occurred at multiple levels, and it involved thematic inductive analysis whereby transcripts were coded using a line-by-line open coding process and constant comparison process as advocated by Corbin and Strauss [26], to develop emergent themes. Ritchie and Spencer’s [27] six-staged (reading, identifying, indexing, charting, mapping and interpreting generated themes) theoretical framework analysis was employed to identify and contextualise the transformative learning aspects of the international rural placement experience. Thematic analysis occurred at multiple levels: (1) motivations for applying, (2) identification of transformative elements, (3) processes of integration of transformative experiences and (4) impact of experience on longer-term learning and clinical practice.

## Results

### Interview participants

The initial survey received responses from 15 participants. Twelve (7 females and 5 males) of these participants were available for the subsequent interviews. Of the 12 interviewees, 7 were medical students (4 females and 3 males), and 5 were graduates (3 females and 2 males) at the time of data collection, four of whom had participated 10 or more years ago. All interviewees participated in the international rural placement between 2002 and 2018. Placement locations for interviewees included Canada (*n* = 4), North Dakota, USA (*n* = 6) and Tennessee, USA (*n* = 2). Participants 13–15 were not interviewed but provided detailed qualitative feedback in their survey responses. Placement locations for these participants included Norway (*n* = 2) and Tennessee, USA (*n* = 1). None of the interviewees were living or working in a rural or remote community at the time of the study, although most (*n* = 10) were living in regional communities, and some had not yet completed specialty training or settled into a permanent position. Participant IDs, linked with their associated quotes, reflect their participant number (P1-15), their academic status at the time of data collection (undergraduate (U) or graduate (G) and placement location.

### Motivations to engage with the application process

The participants found the application process enjoyable and found value in the multi-stage application process. The three-step application process, involving a book/movie review, interview, and researched oral presentation, self-selected a highly motivated group of applicants.

*“I thought that the amount of steps that were required was sort of a good idea, because it meant that unless you really wanted to go on the placement and you were committed to doing all those steps, then it sort of weeded out the people who would have happily just chucked in an application if it took them five minutes. So it took me a day to write my film review and then prepare for the interview and then I also had to make a presentation on an aspect of rural health, but unless you really, really wanted to go on this trip and be a part of the experiences, it got the cream of the crop, in terms of people that were interested and willing to put in the work to get the scholarship.”* [P3-U-NORTH DAKOTA]

Two themes were identified in relation to participants’ motivation: (a) experiencing eye-opening and culturally rich difference and (b) once in a lifetime opportunity.

- *(a).* *Experiencing eye-opening and culturally rich differences*

Most motivations centred around the key theme of “different.” Participants were motivated by the opportunity to travel overseas, to experience a different country/region, different culture, different people, different climate and different healthcare system. Many reported that learning about and experiencing a different culture was a key motivator for applying. For some, this was an interest in learning more about a different indigenous culture. Others also saw this as an opportunity to learn from people from another cultural perspective.

*“But being able to go to a different country where there's different cultures, different expectations of health, different views on health is very, I think, enlightening and eye-opening. So, I think one of the primary motivating factors is being an eye-opening culturally rich experience where you can have a broader view of the world outside. It definitely makes you more tolerant, accepting, sensitive of other people's views, rather than being a bit closed minded.”* [P7-G-TENNESSEE]

*“Having never been overseas, I was very, very young and very inexperienced, I guess. I certainly hadn't travelled anywhere. So, the main thing was, just the idea of being able to travel overseas sounded really amazing and to experience something that was so different, especially climate-wise, going from somewhere like Townsville. I'd never seen snow in my life, so the idea of going to North Dakota in winter and having a white winter, that was quite appealing.”* [P10-G-NORTH DAKOTA]

*“It was just an opportunity for me to go and experience something completely different and see a different side of the world. I was really excited to see how the health and the health studies that I do here, and the studies regarding social circumstances surrounding health, translated to another country that had unique circumstances of its own. Yeah, that was probably what motivated me the most.”* [P2-U-CANADA]

- *(b).* *Once in a lifetime opportunity*

The fact that this placement included a fully funded bursary was another key motivator for many. The uniqueness of the opportunity was considered a “once in a lifetime opportunity” [P12] and an “adventure that needed to be had” [P9]. The belief in the importance of taking up available opportunities motivated the participants to apply for the scholarship.

*“I worked at a CD store, making like $13 an hour. I would never have been able to afford it. My parents wouldn't have been able to afford it either. The financial assistance was the only reason I could go.”* [P9-G-NORTH DAKOTA]

*“I can't understate [laughs] that it was also just a bit of, wow, an overseas trip that I couldn't otherwise afford and an opportunity to see a different part of the world. It was kind of just what I ended up reflecting about, knowing that I had the privilege of getting this prize and therefore I needed to take every opportunity to get out of it what I could.”* [P8-U-CANADA]

### Disorientating dilemmas that created transformative experiences

Some elements of the placement experience presented disorientating dilemmas that were identified to have transformative impacts on the participants. The transformative experiences can be described in terms of a trigger that challenges a person’s previous perception/view/understanding causing mental processing that causes a shift in perceptions/understandings leading to a new aspect/perception/understanding. Three themes were identified in relation to major situations that triggered deep reflections and shifts in perceptions. These were: (a) outsider view allows reassessment of one’s own country, (b) being open-minded to enjoy good company and (c) isolation experiences ginger desire to right health wrongs.

- *(a). *Outsider view allows reassessment of one’s own country

Participants who went to Canada reported that the placement was light clinically, but heavy on Indigenous (First Nations) cultural aspects and social determinants of health (Indigenous and people of low socioeconomic status). This group of participants reported that they had had various prior experiences/knowledge about Australian Indigenous people’s health care, and social determinants of health (broadly) at the university. However, experiencing health care within a Canadian Indigenous peoples context, gave them new insights and better understanding of the cultural differences and challenges. Participants talked about how being taken out of their usual routine in Australia to see the conditions of First Nations peoples helped them understand the dire situation of Indigenous Australian healthcare.

*“I think there are similarities to the situation. Not necessarily because there's similarities between Aboriginal and Torres Strait Islander culture and First Nations culture [in Canada], [but] because I think they were colonized by the same sort of oppressive system, which creates the similarities. But I think because being from here [Australia], you're so involved in our system, you grew up here, you’re inextricable from the culture. Then you go to a different culture and see a similar thing. You're sort of looking at it with the eyes of an outsider. And I think that makes things clearer. But also, I had experiences there that I haven't had with Aboriginal Torres Strait Islander people in Australia. So, I had more intimate experiences than I probably have in Australia, so that has value as well.”* [P12-U-CANADA]

Those who went to the USA felt there was less emphasis on Indigenous cultural issues, but they gained experience in relation to the different health care/health insurance system. They reported having some awareness of the differences in health care and health insurance in the US from university and movies, etc. However, observing seriously ill patients being turned away because they lacked proper health insurance and finding out how much a medical service would cost a person, even with health insurance (e.g., helicopter ride to nearest suitable hospital), made them value the Australian healthcare system more. This experience gave this group of participants a notion of realism when thinking about “what would be an ideal model of care, so the system is accessible but not abused”. A deeper understanding of the differences in the health care system between Australia (universal – anyone can see a doctor – patients may over-present) and the USA (privatised – costs out-of-pocket to go to the doctor, so patients who need medical attention but can’t afford it may not be seen), provided better clarity, though with fewer linkages to Australian Indigenous healthcare.

*“We had a patient who had a heart attack and so he came into the hospital, and just to send him to a tertiary facility would have costed him so much money. And I was really shocked by that. The doctor was telling me it was in the thousands that he would be paying because he had to get the helicopter service to the tertiary hospital. Yeah, that just blew my mind. Whereas in Australia, obviously, that doesn't happen. Just comparing it to Australia where we have like the Royal Flying Doctor Service, and they don't charge a thing for it. So that was really impactful.”* [P11-U-NORTH DAKOTA]

- *(b).* *Being open-minded to enjoy good company*

All participants had an opinion about whether or not to recommend individual or shared placement opportunities. Most participants favoured having placement partners from the university and many of those who travelled together commented on the importance of having a travel companion for value, security, companionship, and familiarity. They attributed much of the comfort on the placement to having a travel companion with whom to debrief (good company). Another element to living/traveling with another, especially someone you didn’t know well before, was the opportunity to develop close social skills, which can be used in both one’s personal and professional lives. For example, one participant discussed his experience of traveling with another student, and how they both were cautious/mindful of the other’s opinions early on. This caused shifts in the ways in which he relates to other people in the form of developing tolerance and understanding of others’ opinions, being more mindful and considerate of the desires and needs of others, and to compromise.

“*I was lucky in that I did get to go with one of my good friends at the time, so we were together for the four weeks. I'm thankful that I had him to kind of relate to. We were definitely debriefing together. Definitely sending people in pairs is a good idea.*” [P8-U-CANADA]

*“*I don't think I would have been able to navigate and move easily as a girl alone in a remote country, like a different country, especially not knowing anyone. My travelling companion was a big burly boy, and it was nice to know that he was there, and whatever we did, we were doing together, and I didn't have to worry as much about my own personal safety.” [P2-U-CANADA]

Participants who travelled alone felt it gave them opportunities to live with host families that they did not know well, this gave them “good company” and also inspired the development of social skills such as nonverbal communication skills. Interacting with others was an important aspect of the mental processing for most participants. They talked about how the experience taught them about how to live with other people.

*“I think getting to live with a family and they sort of welcome you. I think they basically said, ‘You are part of the family for this month. Whatever you want, whatever you need, it's fine. And equally, we expect you to eat dinner with us, and we expect you to do stuff with us’. That was a pretty immersive experience. I don’t know how you can get that any other way.”* [P1-G-NORTH DAKOTA]

In both cases, close interaction with another person, be it a travel companion, host family or patients, helped the participants learn the importance of avoiding assumptions, being non-judgmental (‘don’t know what they’ve been through’), demonstrating compassion, empathy and kindness.

*“I think I needed a lot more confidence going to North Dakota by myself, and just being open minded as well and getting to know everyone. So, I really had to be open minded, get to know other people, which can be scary sometimes, because they're pretty much like strangers, but you just have to trust the process.”* [P11-U-NORTH DAKOTA]

- *(c). Isolation experiences ginger desire to right health wrongs*

The interview triggered deep reflective moments for participants about the placement experience. Major areas of consideration were personal, clinical/professional, and rural/remote isolation experiences. Drawing comparisons with self, life in Australia and the Australian Indigenous population gave the participants a better understanding of self that included an enhanced sense of independence/confidence, personal growth in embracing personal differences and limitations as it relates to degree of remoteness and independence as well as rural healthcare issues. For example, the experience of living in a very rural/remote area acted as a trigger in relation to the physical and social isolation, and access to supplies. The physical isolation caused a shift in understanding of self: participants left with a better understanding of their personal limitations in terms of the degree of remoteness and where they would like to live in the future. The social aspects of the isolation caused a shift in understanding of the world around them, leaving them with a greater appreciation of what they have in Australia and possibly the importance they place on having access to social support. The limited access to supplies that the rural/remote living offered also triggered shifts in the way they thought about things as it relates to social determinants of health.

*“I have acquired a better understanding of the role of social determinants of health and health outcomes in rural/remote areas. (1) It can be really hard to get access to fresh food living in rural/remote communities – both in Canada and in Australia. (2) The limited access to supplies can influence mental health. (3) There are things related to social determinants of health that you as a doctor cannot control; you just have to work with or around it”.* [P8-U-CANADA]

It also elicited a deeper understanding of the world around them, with greater appreciation and gratitude for what they have. One of the participants reported that by experiencing a world different to her own, she came to find greater appreciation for the things she has in Australia which she had previously taken for granted. Most importantly, the experience brought about thoughts on how they would want to practise medicine and the emergence/development of their professional identity. It elicited the desire to temporarily work in low- and middle-income countries, with organisations such as Médecins Sans Frontières.

*“I struggled a bit because I was in Canada in the middle of winter. It was so dark and so cold and having grown up in Australia where it’s like sunshine for 18 hours a day, and warm; I, struggled a bit more than I thought I would. It definitely made me appreciate where I've grown up and where I live. Not that I didn't enjoy it, but it definitely made me thankful for what I have.”* [P8-U-CANADA]

*“The experiences that I had then have shaped who I am, and they have had an impact on how I practice and what I chose to do subsequently and where I work.”* [P5-G-NORTH DAKOTA]

*“I think it definitely reiterated that I want to work with those people [in underserved communities] and Aboriginal Torres Strait Islander people as well. Try and slightly right some of the health wrongs. And at the time, it definitely showed me that you can live rurally and still have a fulfilling life. I'm not sure if I'm still leaning towards working rurally, but I definitely wouldn't work in a big city and I definitely would want to be involved with Aboriginal Torres Strait Islander patients, wherever I work.”* [P12-U-CANADA]

### Processing transformative experiences

The various triggers led to shifts in the ways in which bursary recipients viewed themselves, others, the world, thought about things, and interacted with others in the world around them through a variety of mental processing techniques. The ways in which the experiences were incorporated into the interviewee’s lives began while still on placement and continued upon their return to Australia, third-year studies, and into the present. Two themes were identified in relation to processing transformative experiences: (a) challenging new experiences through critical self-reflection and (b) recalling memories with significant others.

- *(a).* *Challenging new experiences through critical self-reflection*

While most recipients demonstrated some level of unprompted critical self-reflection, some also reported the utility of keeping a diary or writing a reflective blog after the placement as helpful tools to further facilitate their personal reflection of the experience. Some recipients reported that their home and/or host university requested that they write a reflective blog entry about their experiences for publication on their university website.

*“We had to keep a record basically of our thoughts and reflections from each week. We documented what we learnt, what we'd been challenged by, and what we thought about that we hadn't thought of before. I remember documenting in that diary some of these things, thinking about it and reporting back to the university during the time but also communicating with friends and other students who were having different but also enlightening experiences in other places.”* [P5-G-NORTH DAKOTA]

Participants pointed out the value of keeping reflection diaries during the placement and stated that they often revisited the diaries as processing tool. They also talked about how JCU’s curriculum has helped them to build a habit of self-reflection as part of their routine processing of learning. They highlighted how the critical self-reflection process had primed them for life-long learning and fostered transformative experiences.

*“In JCU, they encourage a lot of self-reflection, and there are actually quite a significant number of assessment tasks, which are important in sort of focusing on reflection and sort of force you to self-reflect. I was actually reading one of my reflections on my rural experience, just to jog my memory of those specific examples of the ones that I gave you tonight. Yeah, being able to sort of self-reflect through writing and assignments during university, but also, as you experience new experiences, you always compare that with your previous experiences as well - how those new experiences are either challenged or shaped by your previous experiences.”* [P7-G-TENNESSEE]

- *(b)*. *Recalling memories with significant others*

While on placement, important others who helped to facilitate the processing of the experience included the people they met on placement, the family and friends back in Australia with whom they kept in touch while on placement, and where relevant their fellow JCU travel companion.

*“XXX and I have kind of lost touch now but even in the years afterwards, we'd have lots of funny memories together and jokes about it, even though we weren't close and we weren't even part of the same friend group. I think it was a kind of nice connection to have with him when we came back.”* [P2-U-CANADA]

Upon returning to JCU, recipients are asked to give a number of presentations to potential future bursary applicants. Each of these experiences, even participating in the interviews for this study all offered opportunities for further reflection, processing, and integration.

*“I did those blog posts while I was over in Canada, which was a good opportunity to reflect on what I'd been learning and what I was enjoying. Then chatting to the girl I went with about the placement was another opportunity. Then I just guess I continued to reflect with friends and family when I returned, just chatting to them about my experiences of what I found over there. Yeah, that was - and then also interviewing. So I interviewed for the panel for the selection of the students to go the following year, so that was my other opportunity where I guess it reminded me of going on the trip and stuff and just talking to people there about what it was like.”* [P3-U-NORTH DAKOTA]

When memories of the placement experience were triggered in other areas of their lives, these offered new contexts to further process and integrate their learning outcomes from the placement experience.

*“I'm surprised how often it comes up and comes to mind [laughs], to be honest. So there'll often be things that I'll say, oh, when I was in North Dakota, you know, actually being this focus of reflecting on [this indistinct thinking] that made me realise, well, that was nearly 20 years ago, and I still think about so many of the experiences that are still [indistinct] to this day. I still bring that [experience] up with patients. I still talk to the students about it. You know, I still talk to the kids about it and say, oh, oh, when we were in North Dakota or when Dr Kratcha did this, you know. They probably get sick of hearing me talk about it but it comes up surprisingly often. The experiences that I had then have shaped who I am and they have had an impact on how I practice and what I chose to do subsequently and where I work. Yeah, so because of that it does come up quite often, actually.”* [P5-G-NORTH DAKOTA]

### Impact of the placement experience on longer-term learning and clinical practice

The various triggers had significant impact on the bursary recipients and opened their eyes to new horizons and inspired life-long learning opportunities. The ways in which the experiences were incorporated into the interviewees’ lives began while still on placement and continued upon their return to Australia, including from third year of study to graduation, and into the present. The experience opened the participants’ eyes to career opportunities that they had not previously considered. It also emphasised the value of rural practice and interactions with role model clinicians increased their passion to go rural. Four themes were identified: (a) inspirational new horizon, (b) navigating/ embracing challenges to gain respect and trust, (c) role model for life-long learning and (d) confidence to try new ways of doing things.

- *(a).* *Inspirational new horizon*

The international rural placement allowed most of the bursary recipients to observe a range of medical practice in rural settings, creating a lasting impression of the value of rural generalist practice. The prospect of serving a whole community in a range of medical disciplines as a rural generalist was considered to be both fulfilling for the doctor and beneficial to the community. However, other interviewees reported that observing several specialists working in rural communities broadened their prospects to the possibility of working as a specialist in a rural setting.

*“So I guess I had always thought about rural generalist when I was applying to medicine as well, and I heard about it. But seeing a doctor, who pretty much was a rural generalist, where he was able to do GP work but also work in the ED and the hospital, and do procedures like a gastroenterologist would do, it made me very excited about that pathway as well, and I could see how in the future if I was in a rural location, how I could be a rural generalist as well, or the things that I could do. So I think I realised the real value in becoming a rural generalist and really serving the community in multiple ways and not just having one specialist for obstetrics, one specialist for GP - yeah, I could see the value that a rural generalist has in the community.”* [P4-U-NORTH DAKOTA]

*“It was probably seeing how a specialist was working in a rural place, like the paediatrician. But also, a generalist, the family doctor, was also working in a similar place. So it showed me that it didn’t really matter so much what you did in your career, it just mattered that you had a passion for doing what you do in a rural area. So it showed me that you could do both a specialty like paediatrics or a generalist specialty like family medicine in the same place. Essentially, they were in the same town. So it sort of opened my eyes to the fact that it didn't just have to be generalists working in a rural area.”* [P6-U-TENNESSEE]

- *(b).* *Navigating challenges to gain respect and trust within the community*

In addition to shaping the career direction of many participants, the experience also provided them with experiences that challenged their thinking.

*“I think it demonstrated to me that I would like to be challenged in the same ways I was on placement when I was put in cultural situations that are different to my own and I'm forced to learn how to navigate within those and gain respect and trust within a community; and how to be culturally respectful, and I'm going to use them in the future if I want to do rural and remote medicine.”* [P2-U-CANADA]

- *(c).* *Role models for life-long learning*

Working closely alongside role model clinicians inspired the interviewees to pursue work in rural settings and/or undertake further specialisation.

*“Well, I chose to follow a fairly similar training pathway, so I joined the same pathway that Lynn Kratcha had been on years before. I also went down the Australian GP Training pathway, the rural branch of the Australian rural GP training pathway. Then with a special interest in obstetrics and being involved in that, as he had been.”* [P5-G-NORTH DAKOTA]

*“That experience I think has put me in good stead for doing more rural activities in Australia, examples being last year I did about nine months in Mount Isa. I've done - I've preferenced a lot of really rural places, like Longreach, for fourth year, and I've really tried to get into it. I think that was sparked by Tennessee and having that experience and being lucky enough to be given that opportunity really made me feel like I should be putting everything I have into trying the rural practice area and giving it a go and actually seeing if it will work. So in that respect, I am interning in Toowoomba next year. It renewed my passion for living in a rural community in the community sense as well, in that you get to know everyone in the town and you are known yourself as the student, or as whatever you are in the town. So I've made it a big goal of mine in the future. Whether or not I end up working completely in a rural town or not, I want to be doing some sort of work in a rural area.* [P6-U-TENNESSEE]

Interviewees also reported that this aspect of the experience motivated them to continue their studies on return to Australia and fostered their life-long learning.

*“No issues. If anything, I was very excited. I had even more interest in now learning other new skills, and I was just more excited to keep practising my skills and acquiring the knowledge to then understand certain procedures and things like that. So I was actually more excited about coming back to classes. I think I maybe wanted to finish medicine as quickly as possible, so I could do what [the doctor I worked closely with on the placement] does [laughs]”.* [P6-U-TENNESSEE]

*“I guess you could say that I was reinvigorated to study medicine because, you know, in the early years, we don't do much clinical stuff, it can all seem a bit pointless and fuzzy sometimes, all the study without any patient contact. So there was definitely some inspiration that made it easy to go back and study.”* [P12-U-CANADA]

- *(d).* *Confidence to try new ways of doing things*

Exposure to clinical experience so early in their training, and encouragement from role-model clinicians, also built confidence in the bursary recipients. Upon returning to Australia, and their undergraduate studies, they were able to practise and apply the skills and knowledge obtained from the experience.

*“It gave me confidence because we were doing you know, what I did in North Dakota, I know there was a lot more clinical staff and clinical skills that I learnt compared to my peers. So I felt far more confident that I was doing okay, in medicine.”* [P9-G-NORTH DAKOTA]

*“Lynn taught me this new way of doing it. I still put it into that framework, because it was helpful. It was useful - you know, it was a short summary, and it was really good for recording notes rather than the more longwinded way they were trying to teach us to record for assessment tasks.”* [P5-G-NORTH DAKOTA]

The bursary recipients also reported increased confidence in advocating for their needs in order to maximise their learning during subsequent clinical placements, having been able to learn new skills while on the international rural placement.

*“When I go to placements from that point onwards, I realised what I want out of placements now, just because of what [the doctor I worked closely with on the placement] has taught me. So I’m not afraid of – like now, if the doctor says do you want to have a go, I would say, “Yes, would you be able to supervise me?”. Even if I hadn’t seen anything or haven’t tried it yet, I would let them know, “Is it okay if I watch you do it for the first time?”. Because I’m sort of now using his philosophy to then get the ’most out of my placement ’experiences, and I've found that it's worked wonders.”* [P4-U-NORTH DAKOTA]

Overall, interviewees reported positive associations with the placement. Participants largely described the experience as not only meeting their expectations, but in many cases exceeding their expectations. Indeed, many reported that it was the degree of impact the experience had on their life and the extreme gratitude they have for having had this opportunity that motivated them to participate in this study. One participant reported that the experience was so great it just had to be talked about, which is why they signed up for an interview.

*“It's a pleasure to talk about this because I am so thankful that I had the opportunity. This is something that warrants being talked about. It's something that should continue for other students and I can't praise the program enough for what it gave me.”* [P2-U-CANADA]

## Discussion

The results of this qualitative evaluation study show that early international rural placements for medical students can promote critical reflection on rural health care provision through an immersive experience in a different system where there are both strong similarities and strong differences. This supports the value of Transformational Learning Theory to the concept of providing a ‘different’ experience. The most positive features of the placements were the opportunity to travel internationally, the immersion in another culture, climate, health care system and sometimes the families of rural doctors, provided opportunities to appreciate both similarities to and differences from Australia. Transformational Learning Theory suggests that a number of factors need to be considered when exploring the impact of a transformational learning experience and process. Efficacy depends on the motivation/readiness for change [17]. Participants’ belief in the importance of taking up opportunities and experiencing something ‘different’ motivated them to apply for the bursary, demonstrating an openness, readiness, capacity for critical reflection and self-reflection, positioning participants well for transformational learning.

Fostering a transformative learning experience requires a consideration of the experiential learning context, both in terms of personal and socio-cultural perspectives [9]. The placement experience created an awareness of cultural healthcare system differences. Multiple interviewees discussed how being put in a new environment sparked further consideration or re-evaluation of concepts or social situations they even experienced in Australia. The ‘outsider view’ and isolation experiences were disorientating dilemmas that triggered critical reflection and self-reflection, through which the learner enhanced their understanding of their own frame of reference, its appropriateness to the situation, and shifts in perceptions [20].

Personal context considerations include the type of prior life experience [28], emotional maturity [29, 30], and capacity for critical reflection and self-reflection [9, 15]. Coping and processing strategies appeared to differ greatly between those who had regular access to internet/social media/smart phones and those who participated earlier when technology was less developed. Further, the level and depth of reflection and processing was deeper for those who had graduated and participated more than 10 years ago. Recent participants, some still in medical school, appeared to be still uncovering and discovering the impacts the placement has had and will have on their life.

Still further, the timing, during the junior years of primary medical education, occurs during an important stage of human development and may promote more lasting adjustments to attitudes, beliefs and behaviours. Most interviewees reported excellent social support and strong positive impact on their lives, with happy memories and lasting relationships with hosts. The nature of the social support offered may be key to the transformational experience. The ideal support goes beyond offering comfort; it is support that helps the learner sustain the courage needed to live with the discomfort associated with the disillusionment and to navigate their experience at the ‘edge of their learning.’ This support could come in the form of validation [9]. Participants’ description of their experience of being in ‘good company’ confirm the importance of social support for transformational learning. This opportunity caused shifts in the ways in which they relate with other people, making them more tolerant and understanding [and considerate] of the desires and needs of others. Mezirow argued that rational discourse and trustful communication with others are key elements to foster questioning discussions and the open sharing of ideas that is key to a successful transformational learning experience [18].

Additionally, the utility of keeping a diary or writing a reflective blog during the placement was seen as a helpful tool to facilitate personal reflection of the experience. This confirms that instrumental learning (information about specific steps and direction) is needed to ensure that students have the necessary skills to act on their reconstructed understanding [16]. Additionally, Carter [31] argued that “imaginative relationship (inner-dialogue, meditation) with self was another important relationship type in the transformational learning process. In these personal reflection forms of navigating the disorientating dilemma, other media, such as journaling or writing may play a key role in the transformational process [9]. These sorts of “transformational characteristics [seem to] transcend context” [10, p. 184]. Offering students direct learning experiences that are personally engaging and stimulate personal reflection is a powerful tool for fostering transformative learning.

From the perspective of longer-term education, such a placement may be a valuable opportunity to learn about and understand society and develop as a human being. However, it is not clear that the experiences and improved understanding of rural professional life impacted career choice, in either specialty or location. The location data from the 65 recipients is similar to those of all JCU medical graduates which show higher rural career locations than graduates of other Australian medical schools [32]. Bearing in mind that incentives and support schemes may have value for attraction and retention of healthcare professionals in rural and remote areas [33], it is worth asking whether the rural bursary experience is worth the investment. While it may provide great opportunities for personal development, does it result in enhanced interest and ultimately choice of a career in a rural or remote location? In other words, is it more like a ‘gap year’ experience enjoyed by many students between secondary and tertiary education, where the emphasis is on personal development, or more like a senior clinical elective placement in a specialty of interest (which could be rural medical practice), where the emphasis is on building clinical skills and ‘trying out’ a potential career specialty?

Directing people to work in rural locations and specialties is not necessarily a key performance indicator for the bursary program. While it may foster interest, the insights gained may be equally valuable to people choosing urban practice but regularly dealing with rural doctors and patients. Examples include medical managers, academics (teaching and research) and narrower specialists receiving referrals from rural colleagues. The junior rural bursary experience is likely to lie somewhere in between these two other kinds of experiences, potentially contributing to both personal development and sparking or reinforcing interest in rural health careers. The JCU medical program offers at least three mandatory and more ‘selective’ opportunities to experience rural health care, and the junior bursary may contribute to ‘pipeline’ development. The strong outcomes for rural workforce development are likely to be the result of combining many strategies in selection, curriculum and assessment strategies, rather than a single strategy [34].

### Limitations

This study was conducted within a single medical program that already has a strong focus on the health of rural, remote and indigenous communities, so students may have a higher level of interest in, motivation for and understanding of rural healthcare contexts. Due to the intense competition and application process, successful applicants may be even more interested and knowledgeable and so more prepared for a transformative learning experience. Because of the variation in time since the placement ranged from 1–20 years, opportunities to process and synthesis memories, reflect critically on them and find their meaning may vary. Further, research outcomes may depend on respondents’ capacity to communicate effectively the meaning developed through critical reflection. The small sample size raises the possibility of volunteer bias; those who did not volunteer may not have felt as positive about placement experience. Caution is required in considering relevance of this study to other medical programs, although it may be of value to others in developing similar early international placements. Finally, the bursary may have had additional impacts that are hard to measure and beyond the scope of this study e.g., both unsuccessful applicants and the broader student body may still have benefitted from the experience of peers and surrounding promotion of rural experience. Future research should focus on those at least 10 years post-graduation to explore relative importance of all rural placements experience on career choice – this may indicate the relative importance of the early bursary experience.

## Conclusion

Medical students report positive impacts from the experiences gained from an international rural clinical placement during the junior years. The experiences may adjust thinking, as proposed in Transformational Learning Theory, and appear to contribute to personal psychosocial development. This supports the view that medical programs should consider establishing or maintaining opportunities for international experience of rural professional life in a different health care system. While the impact of international rural placements alone on longer term career choice (specialty and location) remains unclear, at James Cook University, they form part of a consistent, multi-pronged approach to support rural career interest.    

## Supplementary Information

**Additional file 1.** 

## Data Availability

The datasets used and/or analysed during the current study are available from the corresponding author on reasonable request.
