# Supplementary material for: Understanding medical students’ transformative experiences of early preclinical international rural placement over a 20-year period
Source: BMC Med Educ. 2022 Aug 31;22:652. doi: 10.1186/s12909-022-03707-x (PMC9427436; doi:10.1186/s12909-022-03707-x)
Supplement: Supplementary file 1 — Additional file 1. [file 12909_2022_3707_MOESM1_ESM.docx]

# Appendix 1: Interview Guide

# About the participant

Participant demographics and placement details collected in a pre-interview survey.

1. Tell me a little about yourself now. Where are you now, what are you doing?

# Motivations to apply

1. What motivated you to apply?

# Application Experience

Tell me a bit about your application experience.

1. What were the steps?
2. What was the experience of applying like?
3. What motivated you to keep moving through the process?

# Placement experience

1. Tell me about your placement
2. What has been the most memorable or impactful aspect of your placement experience? What made it so?
3. What are the aspects of this program that made it really good?
4. What are the aspects of this program that made it challenging for you? How did you cope or manage?
5. Were there any aspects of the program (inside or outside your placement) that surprised you or challenged you in some way, such as causing a shift in your thinking, expectations, beliefs, the way you viewed yourself, or some aspect of the world around you?
6. Did you find the experience led you to new ways of perceiving, thinking, deciding, feeling, or acting?
7. What was your social support network like throughout this experience? What sort of support did you seek from these individuals?
8. What did you take away from the experience? Were there any lessons learned?

### Return to Australia & studies

1. What was it like returning to Australia after this experience? How was it integrating back into life in Australia (e.g., amongst family, housemates, friends, uni)?
2. How did your experience shape your return to third year studies?

### Shaping your career

1. How did your experience shape your career choice/specialisation?

### Decisions about where to live and work

1. How did the placement influence your decisions about where you would like to live and work and where you are currently living and working?

### Influenced self/personal identity

1. How did your placement experience impact you as a person? (e.g., sense of self, character, identity, etc.)

### Summary/ Synthesis

1. Do you have any additional thoughts about how your Lynn Kratcha placement influenced or shaped your life up until now or into the future?
2. How would you describe these changes within you? For the better, worse, neutral?
3. What is your overall evaluation of your placement experience?
4. Were your motivations for applying and your anticipated experiential outcomes met through the placement experience?

## Recommendations

1. How would you describe your experience to a student considering applying? What recommendations or advice would you give to them?
2. Do you have any recommendations or advice for JCU as they continue to offer this program?
3. What recommendations or advice would you offer another university looking to start a program like this one? What are the key elements of the program they must ensure continue?
